# Middle meningeal artery embolization for chronic subdural hematoma: Interpreting divergent randomized evidence and guiding patient selection

**DOI:** 10.3389/fneur.2026.1929416

**Published:** 2026-08-25

**Authors:** Dongfang Yang, Haibin Zhang, Ji Xia, Baoling Liu, Mei Li

**Affiliations:** 1 The Second Affiliated Hospital of Naval Medical University (Shanghai Changzheng Hospital), Shanghai, China; 2 The Second Affiliated Hospital of Inner Mongolia Medical University, Hohhot, China; 3 Central Hospital of Dalian University of Technology (Dalian Municipal Central Hospital), Dalian, China

**Keywords:** chronic subdural hematoma, clinical indications, complications, embolic materials, evidence synthesis, middle meningeal artery embolization, patient selection

## Abstract

Chronic subdural hematoma (cSDH) is among the most common neurosurgical conditions in adults, with incidence rising steadily in aging populations and with increasing antithrombotic drug use. Burr-hole drainage remains the standard treatment for symptomatic cSDH; however, postoperative recurrence rates range from 8 to 30%. Middle meningeal artery embolization (MMAE) targets the pathological blood supply of the dural neomembrane, intervening at the biological source of hematoma progression and representing a fundamental shift in cSDH management. Between 2024 and 2025, four landmark randomized controlled trials (RCTs)—EMBOLISE, STEM, MAGIC-MT, and EMPROTECT—were published. Their conclusions diverge: EMBOLISE and STEM demonstrated that MMAE significantly reduced treatment failure, whereas MAGIC-MT (*p* = 0.10) and EMPROTECT (*p* = 0.13) did not reach statistical significance on their primary endpoints, despite point estimates consistently favoring MMAE. Unlike published meta-analyses that address the question of whether MMAE is effective overall by pooling effect sizes, this review systematically examines potential sources of heterogeneity across the four RCTs—including primary endpoint definitions, patient population characteristics, embolic materials and technical strategies, the differential impact of open-label design on bias, and embolization timing. Throughout the manuscript, we distinguish three levels of evidence: conclusions directly supported by randomized data (Level A), inferences derived from non-randomized subgroup or indirect comparisons (Level B), and hypotheses grounded in biological reasoning (Level C). On this basis, we propose a multidimensional patient stratification framework incorporating age, antithrombotic status, angiographic classification, and hematoma characteristics, with each recommendation explicitly graded by level of evidence. We emphasize that this framework has not been prospectively validated and is intended exclusively to generate hypotheses for future clinical research, not to serve as a treatment guideline. We further delineate the current clinical indications for MMAE, provide a systematic summary of its complications and safety profile, and identify critical future directions including individual patient data meta-analysis, standardized outcome definitions, and prospective biomarker- and imaging-based validation studies.

## 1 Introduction

Chronic subdural hematoma has an annual incidence of approximately 1.7–20.6 per 100,000 in the general population and up to 129.5 per 100,000 among individuals aged 80 years and older (1–4). Between 2003 and 2016, the incidence of cSDH in the United States more than doubled, and cSDH is projected to become the most common cranial neurosurgical condition in adults by 2030 (1, 3, 4). One-year mortality in patients with cSDH may reach 32% (5), imposing a substantial burden on healthcare systems.

The management of cSDH has long centered on burr-hole drainage (6, 7), which effectively relieves the mass effect of the hematoma but does not address the pathophysiological mechanisms driving hematoma formation and recurrence—including dural neomembrane formation, pathological angiogenesis, and local hyperfibrinolysis (8–10). Consequently, postoperative recurrence rates remain high, with reported ranges of 8–30% (6, 7, 11, 12). For elderly patients, those with coagulopathy, and those requiring ongoing anticoagulation, the superimposition of recurrence risk and surgical risk poses a formidable clinical challenge (13, 14).

In 2000, Mandai et al. (15) first reported the concept of MMAE for cSDH. The biological rationale rests on the finding that cSDH progression depends on persistent leakage from an abnormal capillary network within the dural neomembrane supplied by the middle meningeal artery (8–10, 16); by selectively embolizing MMA branches to interrupt this pathological blood supply, the vicious cycle of angiogenesis–microhemorrhage–inflammatory exudation–hematoma expansion may be fundamentally disrupted (17–19). Over the ensuing two decades, the evidence base for MMAE accumulated progressively, from single-center case series to multicenter observational studies. Kan et al. (20) reported a multicenter experience of 154 embolizations across 14 U. S. centers in 2021, with hematoma stabilization or resolution in 91% and a reoperation rate of only 4.5%. Multiple systematic reviews further supported the safety and efficacy of MMAE (21–24).

In November 2024, two large, independent multicenter RCTs—EMBOLISE and MAGIC-MT—were published simultaneously in the *New England Journal of Medicine* (25, 26). In February 2025, a third major RCT—STEM—appeared in the same journal (27). In July 2025, the fourth large RCT—EMPROTECT—was published in the *Journal of the American Medical Association* (28). In addition, Debs et al. (11) reported an interim analysis of a prospective randomized trial comparing MMAE adjunctive to surgery versus surgery alone for symptomatic cSDH. The conclusions of these four core RCTs are, however, discordant: EMBOLISE and STEM yielded statistically significant positive results showing that MMAE reduced treatment failure, whereas MAGIC-MT (*p* = 0.10) and EMPROTECT (*p* = 0.13)—although their point estimates directionally favor MMAE—did not attain statistical significance on their primary endpoints. This divergence among large, rigorously conducted RCTs is not a weakness of the evidence base but rather an opportunity to examine how differences in trial design, population, intervention, and endpoint selection may have shaped the observed outcomes. Shankar et al. (29) were among the first to question, “Do we have sufficient evidence?” At least eight meta-analyses published rapidly thereafter (30–37) have, through pooled effect estimates, consistently confirmed a net benefit direction for MMAE, yet none have addressed in depth a core question: why did four rigorously designed RCTs reach different statistical conclusions?

### 1.1 Literature identification approach

This review is a narrative review employing a purposive literature identification strategy. The four core RCTs—EMBOLISE, STEM, MAGIC-MT, and EMPROTECT—serve as the primary objects of analysis. Meta-analyses were included if they explicitly incorporated at least two of the aforementioned RCTs and were published in peer-reviewed journals. Ancillary literature (pathophysiology studies, embolic material comparison studies, patient subgroup analyses) was selected on the basis of direct relevance to the heterogeneity analysis framework applied to the four RCTs. The interim analysis of a prospective randomized trial by Debs et al. (11) (*n* = 35) was not incorporated into the core stratified analytical framework of this review owing to its single-center design, limited sample size, and interim nature, but is referenced as supplementary supporting information where relevant. This review represents a non-systematic synthesis of the literature and carries an inherent risk of citation bias.

This paper systematically examines the factors potentially contributing to the heterogeneity of results across the four RCTs—spanning primary endpoint definitions, patient population characteristics, embolic materials and technical strategies, the differential impact of open-label design on bias, and embolization timing—and, on this basis, proposes a precision patient selection framework stratified by level of evidence. A critical distinction is maintained throughout: conclusions directly supported by randomized comparisons (Level A) are explicitly separated from inferences based on indirect evidence (Level B) and from hypotheses grounded in biological reasoning (Level C). In addition, this review provides a structured summary of current clinical indications for MMAE and a systematic appraisal of its safety and complications profile, and identifies priority areas for future investigation including individual patient data meta-analysis, standardized outcome definitions, and prospective biomarker- and imaging-based validation studies.

## 2 Pathophysiology of chronic subdural hematoma

### 2.1 From Virchow to modern molecular pathology

In 1857, Virchow described cSDH as “pachymeningitis hemorrhagica interna,” positing inflammation as central to the disease process (17). Modern molecular and cellular investigations have redefined cSDH as a dynamic inflammatory-angiogenic disease (8–10). Key steps in the pathological cascade include: fibroblast activation following injury to the dural border cell layer, with collagen synthesis via the TGF-β1/SMAD signaling pathway leading to formation of a fibrotic neomembrane encasing the hematoma (8, 10); markedly elevated VEGF concentrations in hematoma fluid relative to peripheral serum, driving the formation of abundant, structurally abnormal, highly permeable capillary networks within the neomembrane (16); and excessive local activation of the fibrinolytic system (tPA/uPA) alongside matrix metalloproteinase (MMP-2, MMP-9)-mediated degradation of the vascular wall, entrapping the hematoma in a self-perpetuating cycle of persistent leakage and expansion (8–10). Trieu and Thomas (8), in a 2026 review, further elaborated on the roles of age-related immune dysregulation, HIF-1α signaling activation, and impaired meningeal lymphatic drainage in sustaining the disease. Arunachalam Sakthiyendran et al. (10), in a 2025 review, comprehensively examined the “membrane-driven” model, emphasizing the neomembrane as a central therapeutic target.

### 2.2 The central position of the middle meningeal artery in the cSDH pathological circuit

**Figure 1:**
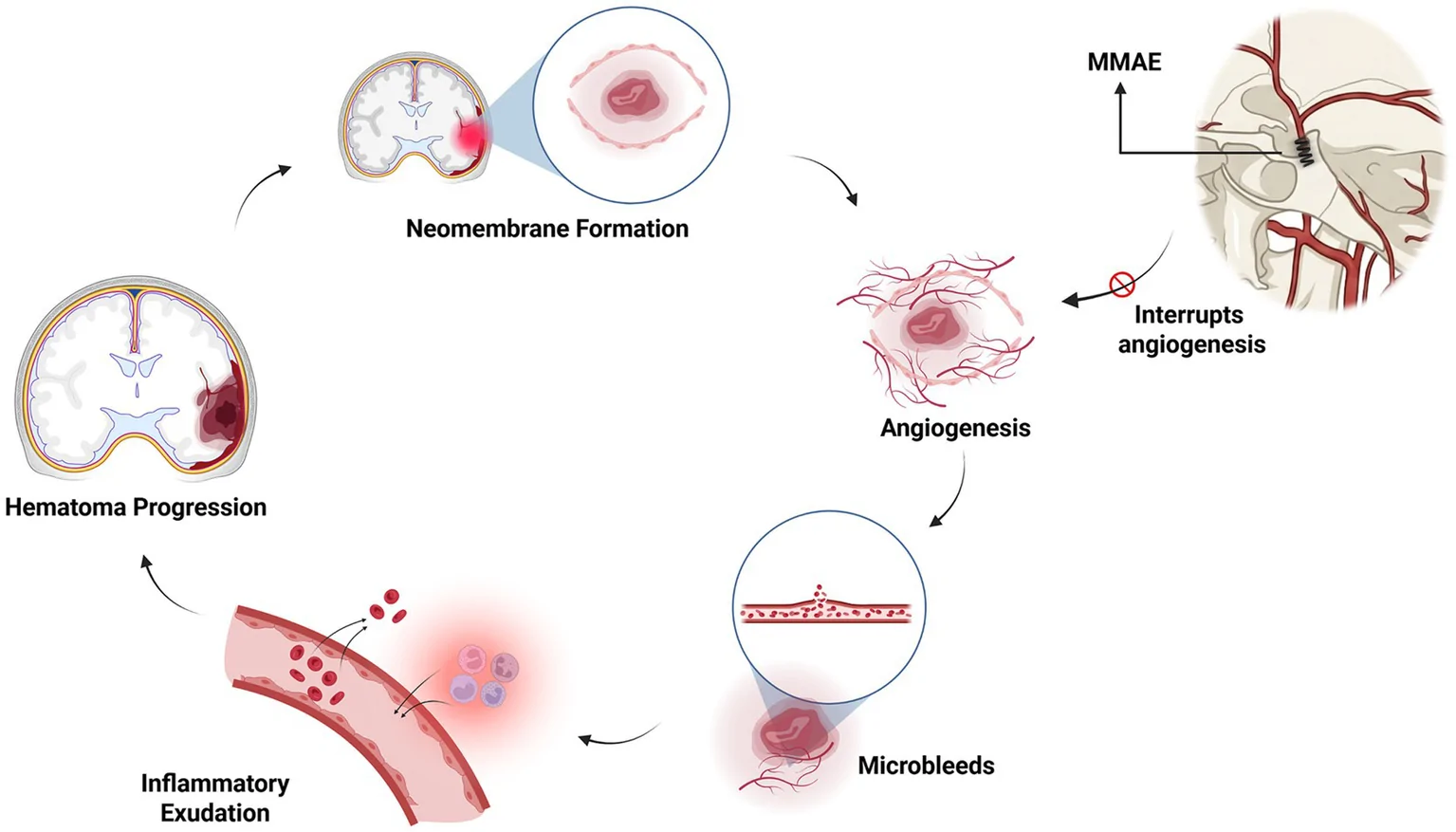
Schematic of cSDH pathophysiological mechanisms and the MMAE intervention target. The vicious cycle driven by the dural neomembrane: Neomembrane formation → pathological angiogenesis → microhemorrhage → inflammatory exudation → hematoma expansion → further neomembrane activation. MMAE blocks this cycle at the middle meningeal artery level by interrupting the arterial supply that sustains pathological angiogenesis within the neomembrane.

The middle meningeal artery is the principal arterial supply of the dura mater. In the cSDH state, MMA branches become markedly dilated and tortuous; superselective angiography may reveal a characteristic “cotton-wool appearance,” reflecting the abnormal density and permeability of the neovascular network (8, 38). The pathophysiological cascade—neomembrane formation driving angiogenesis, which in turn produces microhemorrhage, inflammatory exudation, and further hematoma expansion—constitutes a self-reinforcing vicious cycle (Figure 1). Kupka et al. (39) proposed a three-tier angiographic classification that captures the degree of neovascular activity within this cycle (Table 1): Type I, normal vascular pattern; Type II, cotton-wool staining without significant MMA branch dilatation; Type III, dense cotton-wool staining with pronounced MMA branch dilatation and remodeling. Type III portends a highly active angiogenic state and higher recurrence risk. This classification was derived from a retrospective analysis of 17 patients; the authors themselves explicitly noted that its “prospective application in large studies is needed for validation purposes” (39)—a limitation that directly informs the discussion of evidence levels for angiographically guided treatment selection (§8.3).

**Table 1:** Kupka angiographic classification system.

Type	Angiographic features	Pathophysiological implication
I	Normal MMA morphology; no cotton-wool staining	Low neovascular activity
II	Cotton-wool staining; no significant MMA branch dilatation	Moderate neovascular activity
III	Cotton-wool staining + marked MMA branch dilatation and remodeling	High neovascular activity; elevated recurrence risk

Adapted from Kupka et al. (39). This classification was derived from a retrospective cohort of 17 patients; the authors explicitly noted that its “prospective application in large studies is needed for validation purposes”—a critical caveat relevant to any clinically-oriented application of this system (see §8.3).

MMAE intervenes in this pathological circuit by embolizing MMA branches to interrupt the arterial supply to the neomembrane, thereby blocking the angiogenesis node of the vicious cycle (Figure 1) (17–19). The biological limitations of MMAE, however, merit equal attention (Table 2). First, the MMA is not the sole source of blood supply to the neomembrane—the dura mater possesses a highly complex collateral network, including trans-midline anastomoses from the contralateral MMA, the meningeal recurrent branch of the ophthalmic artery, the anterior falcine artery, meningeal branches of the occipital artery, and the meningohypophyseal trunk from the cavernous segment of the internal carotid artery (40, 41). Samarage et al. (41) described the “bright falx sign”—embolic material crossing the midline to reach the contralateral dura—developed specifically in response to this anatomical challenge. Case reports have documented paradoxical dilatation of the contralateral MMA supplying collateral flow following unilateral embolization (41). Second, there is currently no validated biomarker that can directly confirm complete, durable occlusion of the neomembrane capillary bed following embolization—post-procedural “disappearance of angiographic staining” is not equivalent to thorough necrosis of the capillary network. Salem et al. (42), in an analysis of 636 embolizations, identified independent predictors of failure, including anticoagulant therapy, hematoma thickness, and bilateral disease. Third, the decision between unilateral and bilateral embolization remains largely guided by operator experience, lacking a standardized framework (43, 44); this inter-operator heterogeneity introduces non-trivial confounding into cross-trial comparisons.

**Table 2:** Biological basis and inherent limitations of MMAE.

Dimension	Established evidence	Unresolved questions
Blood supply sources	MMA is the principal feeding artery of the neomembrane (8, 17)	MMA is not the sole source; the relative contribution of collateral networks cannot yet be quantified (40, 41)
Embolization endpoint	Post-procedural angiographic occlusion of MMA branches (19, 41)	“Disappearance of angiographic staining” is not equivalent to complete capillary bed necrosis (42)
Collateral compensation	The bright falx sign indicates that trans-midline compensation can be covered by embolization (41)	Non-MMA collaterals (e.g., meningeal recurrent branch of ophthalmic artery) cannot be blocked by MMAE (40)
Patient selection	Kupka classification reflects angiogenic activity (39)	Optimal embolization strategy for each angiographic type has not been prospectively validated; classification derived from *n* = 17

## 3 Design and principal results of the four landmark RCTs

A comparative overview of the design features, populations, results, and bias risk profiles of the four core RCTs is presented in Table 3.

**Table 3:** Key characteristics and risk of bias comparison across the four RCTs.

Feature	EMBOLISE (25)	STEM (27)	MAGIC-MT (26)	EMPROTECT (28)
Design				
Journal/Year	*N Engl J Med* 2024	*N Engl J Med* 2025	*N Engl J Med* 2024	*JAMA* 2025
Sample size (randomized)	400	310	722	342
Primary endpoint	Repeat surgery for hematoma recurrence/progression within 90 days	180-day composite*	Symptomatic recurrence/progression within 90 days	6-month imaging recurrence (blinded adjudication)
Intervention				
Embolic material	Onyx (EVOH liquid)	SQUID (EVOH liquid)	Liquid embolic (EVOH-based)	TAGM microspheres (300–500 μm)
Technical strategy	Distal penetration	Distal penetration	Distal penetration	Proximal occlusion
Embolization timing relative to surgery	After randomization	Preoperative	Preoperative (99.6%)	Within 7 days post-surgery
Population				
Surgical patients, %	100%	61%	78.3%	100%
Non-surgical patients, %	0%	39%	21.7%	0%
Results				
Primary endpoint (MMAE vs. control)	4.1% vs. 11.3%	16% vs. 36%	6.7% vs. 9.9%	14.8% vs. 21.0%
*p* value	0.008	0.001	0.10 (not significant)	0.13 (not significant)
Effect estimate	RR 0.36 (95% CI, 0.11–0.80)	OR 0.36 (95% CI, 0.20–0.66)	Difference −3.3% (95% CI, −7.4 to 0.8)	OR 0.64 (95% CI, 0.36–1.14)
Risk of bias				
Blinding design	Open-label	Open-label	Open-label	Open-label, blinded endpoint assessment
Endpoint type	Clinical decision-driven	Composite (clinical + imaging)	Clinical decision-driven	Independently adjudicated imaging (blinded)
Endpoint bias risk†	Moderate	Moderate (lower for imaging component)	Moderate to high	Low to moderate

*Hematoma residual >10 mm, reoperation/surgical rescue, major disabling stroke/myocardial infarction/neurological death.

^†^Bias risk ratings are based on a consensus qualitative judgment by the authors using Domain 4 (“Measurement of the outcome”) of the RoB 2 framework and do not represent a systematic application of the complete RoB 2 tool; they are provided for qualitative reference only. See Table 7 for a detailed bias directionality analysis.

### 3.1 The EMBOLISE trial

Davies et al. (25) conducted a prospective multicenter RCT across the United States and Europe, enrolling 400 patients with symptomatic subacute or chronic SDH warranting surgical evacuation, randomly assigned 1:1 to Onyx liquid embolization plus surgery (*n* = 197) or surgery alone (*n* = 203). Of the enrolled patients, 34% had undergone surgery prior to randomization. The primary endpoint was the proportion of patients requiring repeat surgery for hematoma recurrence or progression within 90 days. Results: the reoperation rate was 4.1% (8/197) in the MMAE group versus 11.3% (23/203) in the control group, yielding a relative risk of 0.36 (95% CI, 0.11–0.80, *p* = 0.008). The rate of embolization-related serious adverse events was 2.0% (4/197), including two disabling strokes. Ninety-day mortality was 5.1% in the MMAE group and 3.0% in the control group.

### 3.2 The STEM trial

Fiorella et al. (27) conducted a global multicenter RCT enrolling 310 patients with symptomatic cSDH. Prior to randomization, the treating physician determined whether the patient would be managed surgically (61%, 189/310) or non-surgically (39%, 121/310); patients were then randomly assigned to SQUID liquid embolization plus standard care (*n* = 149) or standard care alone (*n* = 161). The primary efficacy endpoint was a 180-day composite of hematoma residual >10 mm, reoperation or surgical rescue, and major disabling stroke/myocardial infarction/neurological death. Results: the primary efficacy endpoint occurred in 16% (19/120) of the MMAE group versus 36% (47/129) of the control group, with an odds ratio of 0.36 (95% CI, 0.20–0.66, *p* = 0.001). In key subgroup analyses: in the non-surgical subgroup, event rates were 18.8% (MMAE) versus 55.8% (control); in the surgical subgroup, 13.9% versus 23.4%—directionally consistent but with marked differences in magnitude. The primary safety endpoint (major disabling stroke or all-cause death within 30 days) did not differ significantly between groups (MMAE 3% vs. control 3%).

### 3.3 The MAGIC-MT trial

Liu et al. (26) conducted a multicenter open-label RCT in China, enrolling 722 patients with symptomatic non-acute SDH with mass effect. After the treating physician determined the management strategy—burr-hole drainage (78.3%) or non-surgical management—patients were randomly assigned 1:1 to MMAE plus conventional therapy (*n* = 360; ethylene vinyl alcohol [EVOH]-based liquid embolic) or conventional therapy alone (*n* = 362). The primary endpoint was symptomatic hematoma recurrence or progression within 90 days. Results: the primary endpoint occurred in 6.7% (24/360) of the MMAE group versus 9.9% (36/362) of the control group, yielding a between-group difference of −3.3 percentage points (95% CI, −7.4 to 0.8; *p* = 0.10). Notably, this did not reach the conventional threshold for statistical significance, in contrast to the clearly positive results of EMBOLISE and STEM. Serious adverse events occurred in 6.7% of the MMAE group versus 11.6% of the control group (*p* = 0.02). In key subgroups: in the non-surgical subgroup, progression rates were 8.9% (MMAE) versus 21.8% (control); in the surgical subgroup, 6.0% versus 6.7%. The overall control-group event rate in MAGIC-MT (9.9%) was lower than the recurrence rate range historically reported for cSDH (10–30%), which may reflect the specific risk composition of the enrolled population and the stringency of the “symptomatic recurrence/progression” endpoint definition in the context of Chinese clinical practice; these factors, collectively contributing to a lower-than-expected event rate, may have reduced statistical power [Interpretation based on indirect evidence; see Heterogeneity Analysis section].

### 3.4 The EMPROTECT trial

Shotar et al. (28) conducted a multicenter, open-label, blinded-endpoint RCT across 12 centers in France, enrolling 342 patients who underwent surgery for recurrent cSDH or for a first episode with high recurrence risk, randomly assigned 1:1 to MMAE within 7 days post-surgery (*n* = 171; trisacryl gelatin microspheres [TAGM] 300–500 μm) or standard medical care alone (*n* = 171). The primary endpoint was the 6-month imaging recurrence rate adjudicated by an independent blinded endpoint committee. Results: the primary endpoint occurred in 14.8% (24/162) of the MMAE group versus 21.0% (33/157) of the control group, with an odds ratio after imputation of 0.64 (95% CI, 0.36–1.14) and an adjusted absolute difference of −6% (95% CI, −14 to 2%; *p* = 0.13). The authors explicitly noted that “the direction and magnitude of effect estimates were consistent with other recent trials” (28), though it should be noted that the point estimate in EMPROTECT (OR 0.64) is notably more conservative than that in EMBOLISE (RR 0.36); see §6.1 for a detailed discussion of directional consistency versus magnitude equivalence. Ipsilateral reoperation rates were 4.3% (7/162) in the MMAE group versus 8.3% (13/157) in the control group (*p* = 0.14). Mild and severe embolization-related complications occurred in 1.8 and 0.6%, respectively.

### 3.5 Other prospective comparative evidence

Debs et al. (11) reported an interim analysis of a prospective randomized trial (*n* = 35, single-center) comparing MMAE adjunctive to surgery with surgery alone for symptomatic cSDH, reporting that MMAE adjunctive to surgery was superior to surgery alone on overall outcomes. The limited sample size, interim nature, and single-center design of this study constrain its independent evidentiary weight; it was therefore not incorporated into the core stratified analytical framework of this review. Its direction of benefit in the surgical adjunct setting—MMAE plus surgery outperforming surgery alone—is, however, consistent with the direction observed in that setting in EMBOLISE and STEM, and serves as supplementary supporting information. It should be noted that the publicly available abstract of this interim analysis does not provide outcome data disaggregated by specific endpoint type (e.g., recurrence rate, reoperation rate, functional outcome), and the precise comparability of its findings with the four core RCTs on each endpoint therefore cannot yet be assessed. Additionally, the MEMBRANE trial (Middle Meningeal Artery Embolization for the Treatment of Subdural Hematomas With TRUFILL n-BCA), results of which have been presented but not yet formally published in a peer-reviewed journal, provides further evidence directionally consistent with the four core RCTs; its interim status and unavailability of peer-reviewed data preclude inclusion in the core comparative analysis of this review.

## 4 Clinical indications for MMAE in cSDH

The indications for MMAE in cSDH have evolved from early exploratory use to evidence-supported recommendations, though important gaps remain. Current indications can be organized into three clinical scenarios, with the strength of supporting evidence explicitly noted for each. A structured summary is provided in Table 4.

**Table 4:** Clinical indications for MMAE in cSDH.

Clinical scenario	Indication	Level of evidence	Supporting evidence	Key caveats
Stand-alone MMAE				
cSDH requiring intervention + coagulopathy or antithrombotic therapy with unacceptable discontinuation risk (de novo)	Consider stand-alone MMAE as alternative to surgery	B	EANS/ESMINT/ESNR consensus (45); SVIN Guideline Class IIa (63)	Expert consensus based on biological rationale and retrospective data; no RCT specifically testing this indication
cSDH requiring intervention + coagulopathy or antithrombotic therapy with unacceptable discontinuation risk (recurrent)	Consider stand-alone MMAE as alternative to surgery	B	EANS/ESMINT/ESNR consensus (45)	Same as above
cSDH with substantial mass effect / progressive neurological deficit / threatened herniation	MMAE NOT indicated as replacement for timely surgical decompression	—	ARISE I consensus (46); Bartek et al. (45)	Surgery remains standard of care; MMAE may be considered as adjunct to permit earlier antithrombotic resumption [Level B]
Surgical adjunct MMAE				
Recurrent cSDH, surgically managed	Adjunctive MMAE recommended	A	EMBOLISE (25): RR 0.36, *p* = 0.008; EANS/ESMINT/ESNR consensus (45)	EMPROTECT directionally consistent but not statistically significant (*p* = 0.13); MAGIC-MT surgical subgroup showed no difference (6.0% vs. 6.7%)
First-episode cSDH with high recurrence risk, surgically managed	Adjunctive MMAE recommended	A	EMBOLISE; STEM surgical subgroup directionally consistent	“High recurrence risk” not uniformly defined across trials
First-episode cSDH with low recurrence risk, adequately drained	Individualized assessment	A	Same Level A evidence base	Marginal incremental benefit; larger NNT in this subgroup
Indications under investigation (non-surgical management)				
Minimally symptomatic cSDH; no immediate surgery required	Evidence insufficient for routine recommendation	B	Non-randomized subgroups of STEM and MAGIC-MT; Rojas-Villabona et al. (52)	“Non-surgical” decision not randomized; high risk of selection bias; natural history of control group not well characterized
Advanced age (≥80) + high surgical risk, managed non-surgically	May consider standalone MMAE as hypothesis-generating option	B	Non-randomized subgroups; Abo Kasem et al. (4)	Selection bias cannot be excluded; effect magnitude may be partly or entirely attributable to confounding

Level of evidence definitions — Level A: Direct evidence from fully randomized comparisons. Level B: Evidence from non-randomized subgroup analyses, retrospective observational studies, or indirect comparisons; at risk of selection bias, confounding, and/or confounding by indication. Level C (not shown in this table): Theoretical hypotheses grounded in biological reasoning; not yet validated.

### 4.1 MMAE as stand-alone treatment (alternative to surgery)

The multi-society consensus statement issued by Bartek et al. (45) on behalf of EANS/ESMINT/ESNR/ EuGMS/ESAIC/SINCh identified two specific scenarios in which stand-alone MMAE should be considered [Level B; expert consensus based on biological rationale and retrospective data]:

- *De novo* cSDH requiring intervention, where surgery is precluded by coagulopathy or ongoing antithrombotic therapy with an unacceptably high risk of discontinuation;
- Recurrent cSDH requiring intervention, under the same coagulopathy/antithrombotic constraints.

The Society of Vascular and Interventional Neurology (SVIN) guideline (63) assigned a Class IIa (rather than Class I) recommendation for standalone MMAE, explicitly reflecting the uncertainty of evidence for non-surgical applications. Critically, MMAE is *not* currently indicated to replace surgical evacuation in patients with substantial mass effect causing progressive neurological deficits or threatened herniation, for whom timely surgical decompression remains the standard of care (45, 46). In such cases, MMAE may be considered as an *adjunct* to surgery to permit earlier resumption of antithrombotic therapy [Level B].

### 4.2 MMAE as adjunct to surgery

The ARISE I consensus (46) recommended MMAE as an adjunct to surgical evacuation (Class I, Level A), and the EANS/ESMINT/ESNR consensus recommended adjunctive MMAE for all recurrent cSDH cases managed surgically (45). The strongest randomized evidence supporting this indication comes from EMBOLISE (RR 0.36, *p* = 0.008; (25)), with directional consistency from STEM (surgical subgroup OR directionally consistent; (27)) and EMPROTECT (OR 0.64, *p* = 0.13; (28)).

However, it is important to recognize that the evidence for surgical adjunct MMAE is not uniform. In MAGIC-MT, the surgical subgroup showed no appreciable difference between MMAE and control (6.0% vs. 6.7%; (26)). In the meta-analysis by Gillespie et al. (30), MMAE did not significantly reduce recurrence in the surgical subgroup (RR 0.60, *p* = 0.194). This heterogeneity within the surgical adjunct setting underscores that the benefit of adjunctive MMAE may be modulated by patient and procedural characteristics that have not yet been systematically identified [Interpretation based on subgroup-level comparison, Level B].

### 4.3 Indications under investigation (non-surgical management)

Both STEM and MAGIC-MT enrolled non-surgical patients in whom the treating physician determined that immediate surgery was not required, but these decisions were not randomized. In both trials, the effect of MMAE was substantially larger in the non-surgical than in the surgical subgroups (see §6.3 for detailed discussion and the critical caveat regarding selection bias). The ARISE I consensus acknowledged that MMAE is not currently indicated to replace surgical therapy for symptomatic patients who require surgical management (46). The SVIN guideline (63) assigned a Class IIb recommendation (Level B-NR) for MMAE as a standalone treatment option in patients for whom surgery is contraindicated. Currently, the use of MMAE as primary standalone therapy for patients who *could* undergo surgery but in whom conservative management is chosen remains an area of active investigation rather than established practice [Level B, hypothesis-generating only].

### 4.4 Relationship between trial inclusion criteria and indications

The four RCTs themselves enrolled populations that map imperfectly onto these indication categories. EMBOLISE (25) and EMPROTECT (28) enrolled exclusively surgical patients, generating Level A evidence for the surgical adjunct indication. MAGIC-MT (26) and STEM (27) enrolled mixed surgical and non-surgical populations, with non-surgical subgroup analyses generating Level B evidence. The differences in enrolled populations across trials—and the fact that the non-surgical subgroups in STEM and MAGIC-MT are not directly comparable owing to different selection criteria—themselves constitute an important source of the heterogeneity discussed in §6.3.

## 5 Complications and safety profile of MMAE

A systematic appraisal of MMAE-related complications is essential for balanced clinical decision-making. The available evidence—derived from RCTs, large observational series, and systematic reviews—indicates an overall favorable safety profile, though specific complications warrant attention. A summary of reported complication rates across key studies is provided in Table 5.

**Table 5:** Reported complications of MMAE across key studies.

Complication category	Reported incidence	Source(s)	Comments
Overall complications			
Any complication (pooled)	3.1% (95% CI, 2.0–4.8%)	Shafi et al. (5); *n* = 921	Meta-analysis; neurological + cardiovascular + infectious
Any complication	2.3% (58/2,783)	Omura & Ishiguro (47)	Systematic review; includes seizures, renal dysfunction, transient neurological symptoms
Any complication	~3%	ARISE I consensus (46)	Expert consensus estimate
Stroke			
Embolization-related disabling stroke (30-day)	2.0% (4/197)	EMBOLISE (25)	2 disabling strokes potentially related to embolization
All stroke (MMAE vs. control)	2.8% vs. 2.2%	MAGIC-MT (26)	Comparable between groups
MMAE-related stroke (overall estimate)	<1%	ARISE I consensus (46)	Expert consensus
Major disabling stroke (pooled across 4 RCTs)	No significant difference vs. control	Elfil et al. (32); *n* = 1,680	Few events; unstable estimate
Cranial nerve injury			
Facial nerve palsy (Onyx™ reflux to petrous branch)	1 case reported	MAGIC-MT (26)	Specific to liquid embolic agents with distal penetration
Visual complications			
Vision loss/central retinal artery occlusion	1 case (pooled incidence ~2.6%; wide CI)	Shafi et al. (5)	Via meningo-ophthalmic anastomosis; likely underreported in retrospective series
Vision loss risk (overall)	<1%	ARISE I consensus (46)	May be underestimated due to absence of systematic ophthalmological follow-up
EMPROTECT-specific (TAGM microspheres 300–500 μm)			
Mild embolization-related complications	1.8%	EMPROTECT (28)	
Severe embolization-related complications	0.6%	EMPROTECT (28)	
Octogenarians/nonagenarians			
Overall complication rate (≥80 years)	5.8%	Abo Kasem et al. (4)	IPD pooled meta-analysis; higher than overall population
Inpatient mortality (≥80 years)	1.2%	Abo Kasem et al. (4)	Reflects baseline risk of elderly cSDH population
Major safety endpoints across all 4 RCTs			
Severe adverse events (MMAE vs. control)	6.7% vs. 11.6% (*p* = 0.02)	MAGIC-MT (26)	Favoring MMAE
30-day major disabling stroke or death	3% vs. 3%	STEM (27)	No significant difference
90-day mortality (MMAE vs. control)	5.1% vs. 3.0% (not statistically compared)	EMBOLISE (25)	Numerical imbalance; CI likely overlapping

Complication rates across studies are not directly comparable owing to differences in definitions, ascertainment methods, embolic materials, patient populations, and follow-up duration. The distinction between procedure-related complications, device-related complications, and background disease-related events is not uniformly adjudicated across studies.

### 5.1 Overall complication rates

A systematic review and meta-analysis by Shafi et al. (5) encompassing 921 patients reported an overall pooled complication incidence of 3.1% (95% CI, 2.0–4.8%), with neurological complications at 3.8% (95% CI, 2.6–5.5%), cardiovascular complications at 3.6% (95% CI, 2.4–5.4%), and infectious complications at 2.9% (95% CI, 1.9–4.5%). The earlier systematic review by Omura and Ishiguro (47), covering 2,783 patients, reported a similar overall complication rate of 2.3% (58/2,783), including seizures, renal dysfunction, transient neurological symptoms, and puncture-site hematoma; treatment-related mortality was reported in 2 cases. The ARISE I consensus (46) independently estimated the overall MMAE complication rate at approximately 3%, consistent with the meta-analytic estimates above.

### 5.2 Stroke and major neurological complications

Stroke is the most concerning potential complication of MMAE. In EMBOLISE, two disabling strokes potentially related to embolization were reported (30-day rate 2.0%; (25)). In MAGIC-MT, all strokes were comparable between the MMAE group (2.8%) and the control group (2.2%; (26)). The ARISE I consensus estimated the overall MMAE-related stroke risk at less than 1% (46). Pooled analyses across four RCTs (*n* = 1,680) did not detect a significant difference in major disabling stroke between MMAE and control groups [RR not significant; (32)]. It is important to distinguish procedure-related embolic stroke (caused by non-target embolization via dangerous anastomoses or catheter-induced thromboembolism) from the background stroke risk inherent to an elderly cSDH population with a high prevalence of vascular comorbidities.

### 5.3 Cranial nerve injury

Facial nerve palsy resulting from reflux of liquid embolic agent into the petrous branch of the MMA has been documented. MAGIC-MT reported one case of facial nerve paralysis attributed to Onyx™ reflux (26). This complication is specific to liquid embolic agents that can penetrate distally into small-caliber branches and is less likely with proximal coil or large-particle embolization strategies, though this inference has not been formally tested [Interpretation, Level C].

### 5.4 Visual complications

Vision loss via non-target embolization through meningo-ophthalmic anastomoses to the central retinal artery is a widely recognized theoretical risk of MMAE. Shafi et al. (5) identified one case of visual changes related to central retinal artery occlusion (pooled incidence estimated at 2.6%, though based on very few events and wide confidence intervals). The ARISE I consensus estimated vision loss risk at less than 1% (46). The discrepancy between the recognized anatomical risk and the low reported incidence raises the possibility of underreporting, particularly in retrospective series without systematic ophthalmological follow-up [Interpretation]. Thorough pre-procedural angiographic assessment of dangerous anastomoses—including the meningo-ophthalmic, meningolacrimal, and petrosquamosal branches—and superselective microcatheter positioning distal to these branches are standard preventive measures.

### 5.5 Complications in special populations

Abo Kasem, Hubbard, Isidor et al. (4), in an individual patient data pooled meta-analysis focused on patients aged ≥80 years, reported a complication rate of 5.8% and an inpatient mortality rate of 1.2% in this high-risk group. Transradial access and conscious sedation were associated with procedural safety advantages in octogenarians and nonagenarians, though the comparative efficacy of different access routes for recurrence prevention has not been prospectively evaluated [Level B]. In EMPROTECT, which exclusively used 300–500 μm TAGM microspheres, mild embolization-related complications occurred in 1.8% and severe complications in 0.6% (28).

### 5.6 Complication profiles across embolic materials

Whether different embolic strategies carry distinct complication profiles is a clinically important but unresolved question. The distal penetration strategy (Onyx™, SQUID™, n-BCA) carries a theoretically higher risk of non-target embolization via small-caliber anastomoses, whereas the proximal occlusion strategy (particles, coils) may carry a lower risk of cranial nerve and visual complications but a potentially higher risk of incomplete neomembrane devascularization. Available indirect comparisons have not detected statistically significant differences in safety outcomes across embolic agents (48–50), but these analyses are limited by small event counts, between-study heterogeneity, and confounding by operator experience and center-specific practices [Interpretation, Level B–C]. A dedicated randomized comparison of complication profiles across embolization strategies does not exist.

### 5.7 Summary

The overall safety profile of MMAE is favorable, with severe complications occurring in approximately 2–3% of procedures and an overall complication rate of approximately 3%. The most clinically significant risks are procedure-related stroke, facial nerve injury, and—rarely—visual complications. Patient-specific factors (advanced age, vascular comorbidities, anatomical variants of dangerous anastomoses) and procedural factors (embolic material selection, access route, operator experience) likely modulate complication risk, though the relative contribution of each factor has not been quantified prospectively.

## 6 Multidimensional analysis of heterogeneity across the four RCTs

All four RCTs assessed the efficacy of MMAE in patients with cSDH, yet their statistical conclusions diverged. In the following sections, we examine five dimensions—endpoint definitions, the differential impact of open-label design on bias, patient population characteristics, embolic materials and technical strategies, and embolization timing—to analyze potential sources of this heterogeneity. These analytical dimensions differ in their logical hierarchy: endpoint definitions and trial blinding status represent the most upstream variables (“how trial design influences effect detection”), population characteristics represent a midstream variable (“in which populations is the effect manifested”), and embolic materials and timing represent the most downstream variables (“how the intervention is implemented”) (Table 6). Several of the mechanistic explanations proposed in this section—including the differential effects of endpoint selection, embolization timing, embolic materials, and open-label design on trial outcomes—are based on indirect evidence, cross-trial comparisons, and biological reasoning. They should be viewed as hypothesis-generating observations that require dedicated prospective studies for confirmation, rather than as empirically validated explanations for divergent trial results.

**Table 6:** Logical hierarchy of the heterogeneity analysis across the four RCTs.

Analytical dimension	Logical level	Core question	Explanatory power for heterogeneity
Endpoint definition differences (§6.1)	Upstream (trial design)	Are the four trials measuring the same clinical phenomenon?	High—different endpoints have different sensitivity for capturing the same biological effect [Interpretation based on cross-trial comparison]
Differential impact of open-label bias (§6.2)	Upstream (trial design)	Does knowledge of group assignment bias endpoint ascertainment differentially across endpoint types?	High—endpoint type × bias direction interaction may be an important source of heterogeneity [Methodological hypothesis]
Population differences (§6.3)	Midstream (effect modification)	Does the effect of MMAE vary by patient characteristics?	Moderate to high—surgical/non-surgical subgroup data are consistent but subject to selection bias [Level B]
Embolic materials and technical strategies (§6.4)	Downstream (intervention implementation)	Do different embolization approaches produce different biological effects?	Moderate—no head-to-head comparisons; cross-trial comparisons are indirect inferences [Level C]
Embolization timing (§6.5)	Downstream (intervention implementation)	Does pre- versus post-operative embolization influence efficacy?	Low to moderate—no direct comparative evidence; highly interactive with other variables [Level C]

### 6.1 Differences in primary endpoint definitions

The primary endpoint definitions across the four trials differ substantially, and this represents one plausible upstream factor contributing to the divergence in statistical conclusions [Interpretation based on cross-trial comparison of event rates and endpoint definitions].

EMBOLISE (25) selected “hematoma recurrence or progression requiring repeat surgery,” the definition closest to a “hard” clinical endpoint among the four trials. Its advantage lies in unambiguous clinical relevance and relative immunity to measurement error, but it may underestimate the full biological effect of MMAE—if MMAE suppressed hematoma progression but the patient did not undergo repeat surgery owing to mild symptoms or prohibitive operative risk, the endpoint would not be triggered [Interpretation]. The control-group event rate for this endpoint (11.3%) fell in the middle range across the four trials.

STEM (27) adopted the broadest composite endpoint, encompassing hematoma residual >10 mm, reoperation/surgical rescue, and major vascular events. This design increased sensitivity for detecting the biological effect of MMAE (including non-clinical, imaging-based effects) but also resulted in a control-group event rate (36%) far exceeding those of the other three trials. The clinical significance of the imaging component within the composite may vary by subgroup—for patients who have undergone surgery, the clinical importance of “residual >10 mm” may be lower than for those managed non-surgically [Interpretation].

MAGIC-MT (26) selected “symptomatic recurrence or progression,” with a control-group event rate of only 9.9%, lower than many historical estimates (10–30%). This may reflect the specific risk composition of the enrolled population and the stringency of endpoint adjudication criteria within the Chinese clinical practice context, collectively contributing to a lower-than-expected event rate that may have diminished statistical power [Interpretation based on indirect comparison; the trial’s published power calculation is not directly available for verification in the scope of this review].

EMPROTECT (28) employed purely imaging-based recurrence adjudicated by an independent blinded committee—the most objective endpoint definition among the four trials. The 6% absolute risk reduction (14.8% vs. 21.0%) is directionally consistent with EMBOLISE, but the gap between the anticipated effect size (the trial was powered for a 10% absolute difference) and the observed effect size may reflect differing expectations regarding the efficacy of particulate versus liquid embolic agents [Interpretation], the effect of post-operative timing [Interpretation], or simply a more conservative point estimate in a trial with rigorous blinding of endpoint assessment [Interpretation; see §6.2].

Directional consistency versus magnitude equivalence. Despite differences in endpoint definitions, point estimates from all four RCTs point toward risk reduction. However, it would be imprudent to treat benefit across different endpoint types as simply “directionally consistent”—a 6-month imaging recurrence endpoint from EMPROTECT (*p* = 0.13) and a 90-day reoperation endpoint from EMBOLISE (*p* = 0.008) differ fundamentally in clinical meaning and statistical precision. The consistent direction of point estimates reflects an overall trend in the biological signal of MMAE but should not be interpreted as equivalence of clinical benefit in magnitude or certainty [Interpretation].

### 6.2 Differential impact of open-label design across endpoint types

All four RCTs employed open-label designs (EMPROTECT achieved blinding at the endpoint assessment level). This is arguably unavoidable in clinical studies of MMAE—the embolization procedure itself cannot be blinded to the operator. The impact of open-label design on bias is, however, not homogeneous; rather, it depends on the type of primary endpoint and the manner of its ascertainment.

For the “repeat surgery required” endpoint of EMBOLISE (25): the decision to reoperate is made by the treating team, who are aware of the treatment assignment. Two opposite directions of bias are possible—if the treating team believes that MMAE has provided additional protection, they may manage residual hematoma in the MMAE group more conservatively (underestimating the MMAE effect); conversely, heightened vigilance toward the MMAE group may lower the threshold for reoperation (overestimating the MMAE effect). The “symptomatic recurrence/progression” endpoint of MAGIC-MT (26) shares the same bias structure, and its greater reliance on subjective clinical judgment may place it at higher risk of bias [Interpretation].

For the “independently adjudicated imaging recurrence” endpoint of EMPROTECT (28): although the embolization procedure is not blinded to the operator, the primary endpoint was adjudicated by an independent committee under blinded conditions. This represents the most rigorous bias control among the four trials—corresponding to a low risk of bias for Domain 4 (“Measurement of the outcome”) of the RoB 2 framework.

The composite endpoint of STEM (27) includes an imaging component (residual >10 mm) and a clinical component (reoperation/rescue): the imaging component carries a lower risk of bias, but the clinical component shares the bias structure of EMBOLISE.

A qualitative assessment of the differential bias risk across the four trials’ primary endpoints is provided in Table 7.

**Table 7:** Risk of bias assessment for each trial’s primary endpoint under open-label design.

Trial	Primary endpoint	Bias risk*	Direction of bias	Potential impact on effect estimate
EMBOLISE (25)	Repeat surgery required	Moderate	Uncertain (may over- or underestimate)	Effect size may be inflated or attenuated by bias
STEM (27)	Composite (imaging + clinical)	Moderate	Lower for imaging component; uncertain for clinical component	Net bias may lie between EMBOLISE and EMPROTECT
MAGIC-MT (26)	Symptomatic recurrence/progression	Moderate to high	Uncertain; substantial subjective judgment component	Effect size may be inflated or attenuated by bias
EMPROTECT (28)	Blinded-adjudicated imaging recurrence	Low to moderate	Minimal (blinded adjudication)	Effect size likely closest to true value [Methodological hypothesis]

*Based on a consensus qualitative judgment by the authors using Domain 4 (“Measurement of the outcome”) of the RoB 2 framework; not a systematic application of the complete RoB 2 tool. The analysis of bias directionality represents a qualitative logical inference [Interpretation based on endpoint-type × blinding-status interaction; not empirically quantified].

#### 6.2.1 Important caveat

The foregoing analysis of bias directionality represents a qualitative logical inference by the authors grounded in the interaction between endpoint characteristics and blinding status [Interpretation based on endpoint-type × blinding-status interaction; not empirically quantified]. Meta-epidemiological studies specifically examining the impact of open-label design on surgical decision thresholds in surgical/interventional trials are currently lacking. This analysis should therefore be regarded as a methodological hypothesis rather than an empirically validated quantification of bias. It offers a dimension for interpreting heterogeneity that published meta-analyses have not yet addressed: the effect size from EMPROTECT (OR 0.64)—with its blinded endpoint adjudication—may, as a methodological hypothesis, lie closer to the “true” effect of MMAE in the surgical adjunct setting than that from EMBOLISE (RR 0.36), but this inference is speculative and remains to be quantified [Hypothesis].

### 6.3 Differences in enrolled populations

The most important population-level difference across the four RCTs concerns the inclusion of patients managed non-surgically. EMBOLISE (25) and EMPROTECT (28) enrolled only surgical patients. MAGIC-MT (26) and STEM (27) enrolled mixed populations (surgical plus non-surgical), and their subgroup analyses consistently reveal a far larger effect of MMAE in the non-surgical than the surgical subgroup. In MAGIC-MT, progression rates in the non-surgical subgroup were 8.9% (MMAE) versus 21.8% (control), whereas in the surgical subgroup the difference narrowed sharply to 6.0% versus 6.7%; in STEM, 18.8% versus 55.8% in non-surgical patients versus 13.9% versus 23.4% in surgical patients (30).

This pattern suggests a biological interpretation: it is biologically plausible that, for patients who have undergone effective surgical decompression—once mass effect has been relieved by drainage—the metabolic demand of the neomembrane vascular network is reduced, which may attenuate the incremental benefit of blocking its blood supply via MMAE [Interpretation based on biological reasoning, not directly demonstrated]. Conversely, for non-surgically managed patients, MMAE represents the sole intervention directly targeting the disease-driving mechanism, and the effect may consequently be larger [Interpretation].

#### 6.3.1 Key methodological limitation

In all four trials, the decision for “non-surgical” or “conservative” management was not randomized; it was made by the treating physician prior to randomization (26, 27). Patients deemed “not requiring surgery” likely represent a highly selected group with milder disease, smaller hematoma volume, or a different prognostic trajectory. Both Gomez-Paz et al. (51) and Rojas-Villabona et al. (52) have noted the limited evidence and the difficulty of excluding selection bias when MMAE is applied to minimally symptomatic patients. Gillespie et al. (30), in their meta-analysis, explicitly cautioned about residual confounding in the non-surgical subgroup analyses.

In summary: the prominent signal of benefit in the non-surgical subgroup carries important clinical hypothesis-generating value, but its effect magnitude may be partly or entirely explained by selection bias [Interpretation]. This signal should be understood as an effect-modification hypothesis—that disease severity may modify the magnitude of MMAE’s effect—rather than as established evidence of efficacy [Hypothesis, Level B].

### 6.4 Embolic materials and technical strategies: the “material determinism” hypothesis

EMBOLISE employed Onyx (25), STEM employed SQUID (27), and MAGIC-MT employed an EVOH-based liquid embolic (26)—all three representing a distal penetration strategy; EMPROTECT employed 300–500 μm TAGM microspheres (28), representing a proximal occlusion strategy. These different embolization strategies embody two distinct philosophies for intervening on the pathological vascular network of cSDH: pursuit of thorough, casting-type occlusion of the terminal capillary bed (distal penetration), versus reduction of MMA perfusion pressure to indirectly suppress neomembrane blood supply (proximal occlusion) [Interpretation].

#### 6.4.1 Evolution of embolic materials and comparative evidence

Embolic materials for MMAE have evolved from particles to liquids to combination strategies Tudor et al., (62) (Table 8). Ellens et al. (48), in a meta-analysis encompassing 1,134 patients (PVA, *n* = 786; n-BCA, *n* = 167; Onyx, *n* = 181), found no statistically significant differences across embolic agents in recurrence (*p* = 0.71) or surgical rescue rates (*p* = 0.89). Gupta et al. (49), in a systematic review and network meta-analysis specifically comparing Onyx, Squid, and n-BCA, found comparable efficacy and safety among the three, with only a modest advantage for n-BCA over Onyx with respect to all-cause mortality. Salem et al. (50), in a multicenter propensity-score-matched analysis of 1,070 cases, likewise found no clear superiority of one material over another. Hoffman et al. (53), in a systematic review of 346 coil embolization cases, reported a 2.6% surgical rescue rate, comparable to that of liquid embolic agents. The “sugar rush technique” described by Nakagawa et al. (54) and the “bright falx sign” described by Samarage et al. (41) represent the ultimate technical expression of the distal penetration strategy, but their incremental clinical value has not been quantified in prospective comparative studies.

**Table 8:** Overview of embolic materials and strategies for MMAE.

Embolization strategy	Representative materials	Mechanism	Theoretical advantages	Theoretical limitations	Evidence status
Proximal particle embolization	PVA particles, TAGM microspheres	Occlusion of MMA main trunk and proximal branches; reduced perfusion pressure	Low cost; technically simple; repeatable after recanalization	Inadequate distal penetration; risk of late recanalization	EMPROTECT used TAGM (*p* = 0.13); abundant observational data
Proximal scaffold occlusion	Coils	Mechanical occlusion of MMA main trunk	Precise, controllable; low radiation; low recanalization rate	May provide incomplete coverage when used alone	Retrospective case series (*n* = 346; rescue rate 2.6%) (53)
Distal liquid penetration	Onyx, SQUID (EVOH-based); n-BCA (cyanoacrylate)	DMSO/polymerization-driven casting occlusion of terminal capillary bed	Distal penetration; durable occlusion	Technically demanding; high cost; risk of non-target embolization	EMBOLISE (Onyx, *p* = 0.008); STEM (SQUID, *p* = 0.001); MAGIC-MT (EVOH-based liquid embolic, *p* = 0.10)
Combination strategy	Particles + coils / Liquid + coils	Proximal scaffold + distal penetration	Theoretically optimal; adjustable and controllable	Technically complex; lacks standardization	Selected center practice; supported by non-randomized data (41, 54)

No head-to-head RCT comparing embolization strategies exists. All cross-strategy comparisons are indirect and susceptible to confounding by center effects, operator experience, and patient selection differences [Level C for strategy superiority claims].

#### 6.4.2 A critical appraisal of the “material determinism” hypothesis

A hypothesis grounded in physical reasoning posits that the superior distal penetrability of liquid embolic agents partly explains why EMBOLISE and STEM yielded statistically significant results while EMPROTECT did not [Hypothesis]. This hypothesis faces several constraints. First, none of the aforementioned indirect comparisons have detected statistically significant differences among materials (48–50). Second, MAGIC-MT also used an EVOH-based liquid embolic (distal penetration strategy), yet its primary endpoint was not statistically significant, weakening the unidimensional explanatory power of “material determinism.” Third, cross-trial indirect comparisons are highly susceptible to center effects (French particle-based centers versus U. S./European Onyx/SQUID centers), operator experience differentials, and patient management pathway variations—confounders that cannot be excluded. In the absence of a prospective, head-to-head RCT directly comparing different embolization strategies, differences in embolic materials should be regarded as one of the possible contributing factors to the heterogeneity of results across the four RCTs, but cannot be isolated as a standalone explanatory variable [Interpretation, Level C].

### 6.5 Embolization timing

In STEM, MMAE was performed prior to surgery (27); in MAGIC-MT, 99.6% of burr-hole drainage procedures were performed after embolization (26); in EMBOLISE, 34% of patients had undergone surgery before randomization, with the remainder undergoing embolization immediately after randomization (25); and in EMPROTECT, embolization was explicitly mandated within 7 days after surgery (28). The potential advantage of pre-operative embolization lies in the ability to embolize an intact neomembrane vascular network, avoiding surgical disruption of dural anatomy [Hypothesis]; post-operative embolization faces the challenge that surgery has already altered local hemodynamics [Hypothesis]. The investigators of EMPROTECT also mentioned this temporal factor as a possible explanation in their discussion (28).

Embolization timing, however, is highly interactive with embolic material, patient selection, and endpoint definition across the four trials (e.g., EMPROTECT simultaneously features “post-operative embolization,” “particle embolization,” and “enrollment restricted to surgical patients at high recurrence risk”—three characteristics that differ from those of EMBOLISE and STEM), rendering the isolation of its independent effect impossible at the level of currently available aggregate data [Level C]. No direct randomized evidence currently exists comparing the effects of pre-operative versus post-operative embolization.

## 7 Appraisal of published meta-analyses and consensus statements

### 7.1 Contributions and methodological differences among meta-analyses

Following the publication of the four RCTs, multiple research groups rapidly conducted meta-analyses (30–37). These meta-analyses differ substantially in inclusion criteria, choice of effect measure, approach to heterogeneity, and subgroup analysis strategy, yet their conclusions are highly convergent—MMAE reduces cSDH recurrence or treatment failure (pooled RR range, 0.47–0.65) (Table 9). Two meta-analyses paid particular attention to possible subtle differences among embolic agents: Gupta et al. (49) found a modest reduction in all-cause mortality with n-BCA relative to Onyx, whereas the indirect comparison by Ellens et al. (48) detected no statistically significant difference in efficacy.

**Table 9:** Comparison of characteristics of published meta-analyses related to the four RCTs.

Meta-analysis	RCTs included	Sample size	Effect measure	Special methodological features	Key subgroup findings	Limitations (acknowledged by authors or identified by this review)
Gillespie et al. (30)	3	~1,432	RR	Surgical/non-surgical subgroup stratification	Significant benefit in non-surgical subgroup; non-significant in surgical subgroup	Subgroup analysis based on non-randomized stratification; endpoint definition heterogeneity across three RCTs
de Almeida Monteiro et al. (31)	7	1,623	RR	Trial sequential analysis (TSA)	TSA confirmed sufficient evidence	TSA assumes homogeneous effect population, at odds with documented RCT heterogeneity
Elfil et al. (32)	4	1,680	RR	Mortality as secondary endpoint	Reduced mortality (RR 0.53)	Mortality was a secondary endpoint; few events; unstable estimate
Nie et al. (33)	6	1,481	RR	TSA + surgical/non-surgical subgroups	MMAE + surgery reduced failure rate (RR 0.55)	Included non-RCT evidence; high heterogeneity
Papageorgiou et al. (34)	6	1,548	OR	Safety endpoint analysis	NNT = 13	Included non-RCTs; some studies with small sample sizes
Shakir et al. (35)	3	~1,432	RR	Focused on EMBOLISE/STEM/MAGIC-MT	Reduced recurrence (RR 0.50)	Only three RCTs; did not explore sources of heterogeneity
Sattari et al. (37)	9 (incl. Non-RCTs)	1,523	OR/RR	Early landmark meta-analysis	NNT = 7	Predominantly retrospective studies; very high heterogeneity
Kabir et al. (36)	4	~1,700	RR	Dual safety + efficacy endpoints	Confirmed overall direction of benefit	Methodological details await confirmation in peer-reviewed publication
Gupta et al. (49)†	Multi-arm network	—	OR	Network meta-analysis of embolic agents	n-BCA associated with modestly lower mortality than Onyx	Indirect comparisons; substantial between-study differences in patient baseline characteristics

^†^The work of Gupta et al. (49) is a network meta-analysis whose primary objective is the comparison of embolic agents (Onyx, Squid, n-BCA). Its included study set consists predominantly of observational studies and differs fundamentally from traditional meta-analyses in both study population and research question; it should not be interpreted as a parallel validation of the conclusions of the four core RCTs.

The work of Gillespie et al. (30) occupies a special methodological position among the existing meta-analyses: they performed a pooled analysis of three RCTs—EMBOLISE, STEM, and MAGIC-MT (nominal total sample size, 1,432)—with an explicit subgroup stratification by surgical versus non-surgical management. The overall effect approached but did not cross the conventional significance threshold (RR 0.50, 95% CI, 0.23–1.06; *p* = 0.058). In the surgical management subgroup, MMAE did not significantly reduce recurrence (RR 0.60, *p* = 0.194); in the non-surgical management subgroup, it significantly reduced progression (RR 0.36, *p* < 0.001). Gillespie et al. themselves, however, explicitly cautioned that the non-surgical subgroup analysis carries a risk of residual confounding, and that, given the heterogeneity of endpoint definitions, embolic materials, and populations across the three RCTs, the pooled effect estimate under a fixed-effect model may obscure genuine subgroup differences (30).

de Almeida Monteiro et al. (31) expanded the inclusion to seven RCTs with 1,623 patients and, using trial sequential analysis (TSA), confirmed that the accrued evidence had surpassed the required information size. The validity of TSA, however, rests on the assumption that the included trials are drawn from a single underlying effect population—if the four RCTs are not evaluating the same intervention by virtue of differences in endpoint definitions, populations, and materials, then TSA, which assumes a homogeneous effect population, may have issued a premature signal of certainty. This potential methodological limitation has not been adequately discussed in the meta-analytic literature surrounding the four RCTs [Critical perspective grounded in logical analysis by the authors; not derived from a systematic methodological assessment].

It should be noted that the identification and comparison of the above meta-analyses in this review represent a narrative synthesis and may be subject to selective citation bias. The arguments advanced above—concerning the issues left unaddressed by the meta-analyses and the possibility that TSA “may have issued a premature signal of certainty”—should be understood as critical perspectives grounded in logical analysis and comparative reasoning by the authors, rather than as conclusions derived from a systematic methodological assessment. The latter would require a comprehensive search and independent quality appraisal, which lie beyond the methodological scope of this narrative review.

### 7.2 Issues not addressed by existing meta-analyses

Although these meta-analyses have consistently confirmed the direction of MMAE’s benefit, within the analytical framework of this review, they collectively present three issues that have not been adequately discussed in the existing literature—it should be noted that this observation is based on the non-systematic literature identification conducted for this narrative review, and whether these issues are universally present across all published meta-analyses would require confirmation through an independent systematic methodological assessment. First, they “averaged” rather than “dissected” heterogeneity. All analyses report the I^2^ statistic, but none decomposed the cross-trial heterogeneity into components attributable to endpoint definitions, population characteristics, material differences, and timing differences, respectively. Second, they were unable to distinguish “statistical significance” from “clinical significance.” The *p* = 0.10 of MAGIC-MT and *p* = 0.13 of EMPROTECT are absorbed into the pooled effect estimate, and most meta-analyses report a statistically significant pooled *p* value; this, however, essentially “borrows” the statistical power of EMBOLISE and STEM and does not resolve the core question of whether MMAE confers clinically meaningful benefit specifically in the surgical adjunct setting. Third, they may have issued a premature signal of certainty [Interpretation; see §7.1 discussion of TSA]. The pathway to overcoming these limitations lies in individual patient data meta-analysis (IPD-MA), which would enable testing of age, anticoagulation status, and angiographic classification as effect modifiers through interaction analyses (30, 55).

### 7.3 Positions and divergences among consensus statements

The ARISE I consensus (46), published in 2024 and co-developed by academic, industry, and government stakeholders, emphasized MMAE as “a powerful adjunctive treatment” and recommended it for the surgical adjunct setting (Class I, Level A). The multi-society joint consensus issued by Bartek et al. (45) on behalf of EANS/ESMINT/ESNR proposed three clinical scenarios: surgical alternative in the setting of coagulopathy or unacceptable antithrombotic discontinuation risk, surgical adjunct for recurrent cSDH, and general surgical adjunct. The 2025 SVIN guideline (63) assigned a Class IIa (rather than Class I) recommendation for standalone MMAE, reflecting the uncertainty of the evidence for non-surgical applications. Anderson and Smedley (56), in a commentary, posed the question “Is it time for a paradigm shift?” while emphasizing the need for further evidence to refine the applicable patient population (Table 10).

**Table 10:** Comparison of positions on MMAE recommendations across major consensus statements.

Consensus statement	Year	Surgical adjunct MMAE	Standalone MMAE (alternative to surgery)	Recurrent cSDH	Evidence base
ARISE I (46)	2024	Class I, Level A	Not separately graded	Subsumed under surgical adjunct recommendation	EMBOLISE, STEM, MAGIC-MT (data known prior to publication)
EANS/ESMINT/ESNR (45)	2024	Recommended	Recommended (when coagulopathy contraindicates surgery)	Recommended (surgical adjunct)	Evidence synthesis completed before publication of all four RCTs
SVIN Guideline (63)	2025	Recommended	Class IIa (standalone); Class IIb (when surgery contraindicated, Level B-NR)	Recommended	Incorporated complete EMBOLISE, STEM, MAGIC-MT data

## 8 Precision patient selection: stratification by level of evidence

### 8.1 Critical caveat

The stratification framework presented in this section is based on indirect evidence (non-randomized subgroup analyses, retrospective observational studies), post-hoc subgroup findings, and biological plausibility reasoning. It has not been prospectively validated in any study specifically designed to test stratification hypotheses. This framework is intended exclusively to generate hypotheses for future clinical research and to illustrate how multidimensional patient characteristics may interact with MMAE efficacy. It must not be interpreted as a treatment guideline, a validated clinical prediction rule, or a basis for modifying current standard-of-care decisions. Readers are cautioned that when a patient simultaneously satisfies criteria at different evidence levels, clinical decision-making should be guided primarily by the higher level of evidence.

Against a background in which the evidence from four RCTs has not yet coalesced into a unified conclusion, clinical decision-making requires a multidimensional stratification framework more refined than the question “Is MMAE effective?” The framework presented below integrates four dimensions—age, antithrombotic status, angiographic classification, and hematoma characteristics—while explicitly differentiating the strength of the evidence source for each dimension: whether derived from fully randomized comparisons (Level A), non-randomized subgroup analyses or retrospective observational studies (Level B), or theoretical hypotheses (Level C). Each recommendation is accompanied by the appropriate level of certainty (Table 11).

**Table 11:** Evidence-based patient stratification framework for MMAE (graded by level of evidence).

Characteristic combination	Clinical hypothesis to be tested [HYPOTHESIS]	Level of evidence	Nature of evidence source	Key limitations
Surgical adjunct setting				
Recurrent cSDH, surgically managed	Hypothesis: adjunctive MMAE reduces reoperation (supported by Level A RCT data); testable in future trials	A	Fully randomized data + multi-society consensus	EMBOLISE positive; EMPROTECT directionally consistent but not statistically significant
First episode, high recurrence risk, surgically managed	Hypothesis: adjunctive MMAE reduces reoperation (supported by Level A RCT data)	A	Fully randomized data	EMBOLISE positive (RR 0.36, *p* = 0.008); STEM surgical subgroup directionally consistent
First episode, low recurrence risk, adequately drained	Hypothesis: incremental benefit of MMAE in this subgroup is small; larger NNT	A	Fully randomized data	Marginal incremental benefit in this subgroup; larger NNT
Non-surgical management setting				
Advanced age (≥80 years) + high surgical risk	Hypothesis: standalone MMAE may reduce hematoma progression in this subgroup [Level B]	B	Non-randomized subgroup analyses + retrospective data	STEM/MAGIC-MT non-surgical subgroups; selection bias cannot be excluded
Need for early resumption of anticoagulation/antiplatelet therapy	Hypothesis: MMAE may permit safer/faster antithrombotic resumption [Level B]	B	Retrospective studies	Abo Kasem et al. (13); confounding by indication in “resumption” decision
Bilateral disease	Hypothesis: MMAE may be beneficial in bilateral cSDH [Level B]	B	Non-randomized subgroup analyses + observational data	Salem et al. (42) failure predictors; confounding incompletely controlled
Minimally symptomatic; no immediate surgery required	Hypothesis: benefit of standalone MMAE in this subgroup is uncertain [Level B]	B	Non-randomized subgroup analyses	Rojas-Villabona et al. (52); natural history of the control group not well characterized
Embolization strategy selection (based on biological hypotheses)				
Kupka Type III (dense staining + MMA dilatation)	Hypothesis: distal penetration strategy (e.g., liquid embolics) may have greater biological efficacy in highly angiogenic subtypes [Level C]	C	Theoretical hypothesis	Lack of direct evidence correlating post-embolization capillary bed occlusion with angiographic classification; classification derived from *n* = 17; no head-to-head RCT
Kupka Type I (normal vascular pattern)	Hypothesis: proximal occlusion strategy (e.g., coils/particles) may be biologically sufficient in low-activity subtypes [Level C]	C	Theoretical hypothesis	Same limitations as above

Level of evidence definitions: Level A: Direct evidence from fully randomized comparisons; risk of bias controlled through randomization. Level B: Evidence from non-randomized subgroup analyses, retrospective observational studies, or indirect comparisons; at risk of selection bias, confounding, and/or confounding by indication. Level C: Theoretical hypotheses grounded in physical/biological reasoning; not yet validated through prospective clinical investigation; should not serve as the primary basis for current clinical decision-making.

Critical caveat — read before interpreting this table. This stratification framework has NOT been prospectively validated in any study specifically designed to test stratification hypotheses. All Level B recommendations derived from subgroup analyses or observational data are intended solely to inform the direction of future prospective research hypotheses and must not be equated with Level A efficacy evidence confirmed through randomization. Specifically, Level B hypotheses such as “advanced age/high surgical risk → consider standalone MMAE” reflect the effect signal from the non-surgical subgroups of STEM and MAGIC-MT; this signal may be partly or entirely explained by selection bias—namely, patients selected by their treating physicians for non-surgical management inherently possess prognostic characteristics that differ from those of surgical patients. When a patient simultaneously satisfies criteria at different evidence levels, clinical decision-making should be guided primarily by the higher level of evidence. This framework is intended exclusively to generate hypotheses for future clinical research. It must not be interpreted as a treatment guideline, a validated clinical prediction rule, or a basis for modifying current standard-of-care decisions.

### 8.2 Age

Abo Kasem et al. (4), in an individual patient data meta-analysis focused on patients aged 80 years and older, found that this population bears the highest burden of cSDH disease yet was markedly underrepresented in the four RCTs—patients aged ≥90 years were explicitly excluded from some trials. In that study, transradial access and conscious sedation demonstrated advantages in procedural safety among octogenarians and nonagenarians. Procedural safety and MMAE efficacy (recurrence prevention), however, are distinct endpoints—transradial coil embolization under conscious sedation offers advantages in procedural tolerability, but its comparative efficacy for recurrence prevention awaits prospective validation. Consequently, the evidence for benefit from MMAE in elderly patients is graded Level B (derived from non-randomized subgroup analyses and retrospective data), rather than direct evidence from fully randomized comparisons.

### 8.3 Antithrombotic medication management

The high prevalence of antithrombotic drug use among patients with cSDH creates a core clinical dilemma: discontinuation increases thromboembolic risk, whereas continuation may increase rebleeding risk. Abo Kasem et al. (13), in a systematic review and meta-analysis (3 studies; 233 patients), assessed the safety of resuming antithrombotic therapy after MMAE. Key findings include: no significant difference in recurrence between the resumption and non-resumption groups (OR 1.64, 95% CI, 0.45–6.00; *p* = 0.45); a significantly higher rate of thromboembolic complications in the discontinuation group than in the resumption group (12.6% vs. 3.5%); and early resumption (1 week to 1 month) not associated with increased hemorrhagic events but reducing thrombotic risk. These data, however, are derived entirely from retrospective studies (moderate NOS scores), and the decision to resume antithrombotic therapy is itself subject to confounding by indication—clinicians are more inclined to resume antithrombotic therapy in patients deemed to be at lower hemorrhagic risk. None of the four RCTs standardized post-procedural antithrombotic management. Accordingly, the evidence regarding the safety and timing of antithrombotic resumption after MMAE is graded Level B.

### 8.4 Angiographic classification

The Kupka classification (39) reflects the degree of neovascular activity in the disease. A hypothesis grounded in physical reasoning posits that Type III (dense cotton-wool staining + MMA branch dilatation) may have a greater requirement for distal penetration strategies, whereas Type I (normal vascular pattern) may be adequately served by proximal occlusion alone [Hypothesis, Level C]. This hypothesis, however, lacks direct evidence correlating the extent of post-embolization capillary bed occlusion with angiographic classification—no conventional clinical technique currently exists for directly verifying the degree of capillary bed occlusion after MMA embolization; the “disappearance of angiographic staining” on DSA remains the only available intra-procedural endpoint indicator, yet it is not equivalent to complete necrosis of the capillary bed. Consequently, linking angiographic classification to a specific embolization strategy represents biological reasoning (Level C evidence), rather than a validated clinical guideline. It deserves particular emphasis that the Kupka classification was derived from a retrospective cohort of only 17 patients and the authors themselves explicitly called for “prospective application in large studies for validation purposes” (39). None of the four RCTs performed stratified randomization by angiographic classification, implying that the distribution of patients with different angiographic types across trials may be subject to chance imbalances, further obscuring the testability of this hypothesis within the existing evidence base.

### 8.5 Integrated stratification framework

Synthesizing the above dimensions—age, antithrombotic status, angiographic classification, hematoma characteristics, and recurrence status—yields a preliminary patient stratification framework that explicitly distinguishes the strength of the evidence source for each recommendation (Table 11). The framework should be read with the following caveats in mind: (a) all Level B recommendations are derived from non-randomized data and carry a meaningful risk of selection bias, confounding, and ecological fallacy; (b) Level C recommendations represent theoretical hypotheses that have not undergone any clinical validation; (c) the framework has not been tested as an integrated whole in any prospective study; and (d) it is an organizational tool for hypothesis generation that delineates the current boundaries of evidence, not a validated algorithm for clinical decision-making.

## 9 Future research directions

First, an individual patient data meta-analysis (IPD-MA) of EMBOLISE, STEM, MAGIC-MT, and EMPROTECT represents the most powerful available tool for testing age, anticoagulation status, angiographic classification, and hematoma characteristics as effect modifiers through interaction analyses. Unlike conventional aggregate-data meta-analyses, IPD-MA can address the questions that none of the published syntheses have resolved: which patient-level characteristics modify the magnitude of MMAE’s treatment effect, and to what extent do cross-trial differences in endpoint definitions, populations, and interventions account for the observed heterogeneity in trial conclusions? The international collaborative framework called for by Edlmann et al. (55) in 2020 has acquired even greater methodological urgency in the context of the current evidence landscape. The IPD pooled meta-analysis by Abo Kasem et al. (4), focused on patients aged ≥80 years and able to identify predictors of complete hematoma resolution (including the combination of n-BCA with coil embolization), serves as a methodological template for what a comprehensive IPD-MA across all four trials could achieve. An IPD-MA would also yield verifiable candidate variables for an objectively defined criterion of “non-surgical suitability” (e.g., known variables distinguishing surgical from non-surgical management that can be extracted from the published baseline tables of STEM and MAGIC-MT—such as mean GCS, hematoma thickness distribution, and anticoagulant use proportions—may serve as suggestive parameters, though their definitive validation requires individual patient-level analysis). Recognizing that an IPD-MA would require transnational cooperation among the four trial groups and reconciliation of commercial interests—a process likely to require 3–5 years—a more pragmatic near-term operational approach is warranted: professional societies or guideline development groups could adopt an “evidence dossier approach,” in which data from the four RCTs are extracted and presented side-by-side for each endpoint (surgical rescue rate, imaging recurrence rate, functional outcome), accompanied by certainty-of-evidence ratings for each endpoint assigned by independent methodologists using the RoB 2 framework. Such an approach would provide a more transparent and precise current basis for clinical recommendations than the simple pooling of effect estimates, pending the completion of an IPD-MA.

Second, given that existing indirect comparisons have not detected statistically significant differences among embolic materials (48–50), a prospective RCT directly comparing different embolization strategies (distal penetration versus proximal occlusion) is needed, with stratified randomization by Kupka angiographic classification, to formally test the hypothesis that “different angiographic types have differential requirements for embolization strategy.”

Third, given the non-randomized nature of current non-surgical subgroup analyses and their inherent risk of bias, a rigorous trial is needed to compare MMAE as standalone initial therapy against standard non-surgical management (statins, corticosteroids, or close observation) in a randomized design. The eligibility criteria for such a trial should be based on objective indices—such as a threshold for maximum hematoma thickness and a standardized neurological deficit score—rather than relying on the physician’s subjective judgment of “non-surgical” candidacy, as in the existing trials.

Fourth, the marked differences in endpoint definitions across the four RCTs underscore the urgent need for a core outcome set (COS) for cSDH clinical trials. The ARISE I consensus (46) has recommended the development of standardized outcome definitions. A cSDH-specific COS should at minimum include: (a) surgical rescue rate (the hardest and least bias-prone clinical endpoint); (b) standardized imaging recurrence/residual rate (with pre-specified volumetric or diameter thresholds and blinded central adjudication); (c) functional independence at 90 days (mRS score, assessed by blinded raters); and (d) patient-reported outcome measures addressing symptom burden and quality of life. The development of such a COS should follow a formal Delphi consensus process involving clinicians, methodologists, and patient representatives, rather than being proposed by any single research group. The adoption of a COS across future trials would eliminate the endpoint heterogeneity that currently complicates cross-trial comparisons and meta-analytic synthesis.

Fifth, prospective studies incorporating imaging-based and molecular biomarkers as pre-specified stratification variables are needed to test whether these markers can identify differential responders to MMAE. The angiographic classification proposed by Kupka et al. (39)—derived from a retrospective cohort of only 17 patients and explicitly awaiting prospective validation—represents the most immediately testable imaging biomarker. Additional candidate biomarkers include: neomembrane enhancement patterns on contrast-enhanced MRI (e.g., the degree and distribution of “cotton-wool enhancement” as a surrogate for angiogenic activity); DynaCT perfusion characteristics of the dura adjacent to the hematoma; and molecular markers in hematoma fluid (e.g., VEGF, IL-6, and inflammatory cytokine concentration gradients) that may correlate with angiogenic drive and thus with the likelihood of MMAE efficacy. Embedding these biomarkers as pre-specified stratification factors in future RCTs would directly address the patient-selection question that this review has identified as the central unresolved issue in the field.

Sixth, the pathophysiology of cSDH presents multiple targetable nodes (8–10). Statins (57) target inflammatory metabolic pathways, corticosteroids (58) target broad-spectrum inflammation, and bevacizumab (59) targets the VEGF-mediated angiogenic hub. Musmar et al. (60), in a review of combined statin and MMAE therapy, explored the theoretical promise of multi-nodal combined targeting strategies. The DEX-CSDH trial (58), although finding that dexamethasone reduced the need for surgical intervention, was associated with significantly increased adverse events, suggesting a narrow therapeutic window for pharmacotherapy and highlighting the need for carefully designed prospective studies to determine the dosing and timing of combination strategies.

Seventh, the role of MMAE in chronic subdural hygroma—a condition that shares some clinical and anatomical features with cSDH but is characterized by CSF-like fluid accumulation with potentially lower angiogenic activity—remains entirely uninvestigated. The four landmark RCTs did not include hygroma-specific sub-analyses, nor sub-group analyses based on the hemorrhagic versus non-hemorrhagic composition of the subdural collection, and no dedicated prospective study has addressed this question. Whether MMAE confers benefit in patients with chronic subdural hygroma lacking a significant hemorrhagic component is unknown. The hypothesis that MMAE may exert an indirect effect on hygroma via modulation of shared inflammatory and neoangiogenic pathways in the dural border cell layer is biologically plausible but entirely unvalidated [Hypothesis, Level C], and represents an area for future investigation rather than a basis for current clinical decision-making.

## 10 Conclusion

In the field of middle meningeal artery embolization for chronic subdural hematoma, four RCTs have tested interventions that are similar in essence but not identical: in different populations (surgical versus mixed), using different embolization strategies (distal penetration versus proximal occlusion), at different time points (preoperative versus postoperative), and defining different endpoints (reoperation versus composite versus symptomatic versus imaging). Understanding how these differences contribute to the divergence in statistical conclusions—rather than simply pooling effect estimates—is a prerequisite for evidence-based clinical decision-making.

On the basis of a comprehensive analysis of the current best evidence, the following conclusions may be drawn:

First, point estimates from all four RCTs point toward risk reduction. The clinical significance, effect magnitude, and optimal assessment time window of the benefit, however, differ fundamentally across endpoint definitions. The independently adjudicated, blinded imaging endpoint of EMPROTECT (*p* = 0.13) and the clinical reoperation endpoint of EMBOLISE (*p* = 0.008) cannot be regarded as equivalent in statistical precision or clinical meaning. The consistent direction of point estimates reflects an overall trend in the biological signal of MMAE, but should not be interpreted as equivalence of clinical benefit.

Second, in the surgical adjunct setting, the benefit of MMAE is supported by direct evidence from fully randomized comparisons (Level A). EMBOLISE (RR 0.36, *p* = 0.008) and STEM (OR 0.36, *p* = 0.001) both attained statistical significance, and EMPROTECT (OR 0.64, *p* = 0.13) is directionally consistent. In the non-surgical management setting, the signal of benefit from MMAE is more prominent (absolute risk reductions of 13–37 percentage points in the non-surgical subgroups of STEM and MAGIC-MT), but this signal derives from non-randomized subgroup analyses whose effect magnitude may be partly or entirely attributable to selection bias (Level B). The observation that “benefit is greater in non-surgical subgroups” should be understood as a hypothesis awaiting prospective validation, rather than as sufficient evidence for a strong recommendation in favor of standalone MMAE at the present time.

Third, comparative efficacy among embolic materials currently lacks head-to-head RCT evidence. Existing indirect comparisons have, on the whole, not detected statistically significant differences, and cross-trial comparisons are highly susceptible to confounding by center effects and operator experience. The hypothesis that Kupka classification should be matched to embolization strategy is biologically plausible but represents as-yet unvalidated theoretical reasoning (Level C) and should not serve as the primary basis for current clinical decision-making.

Fourth, multidimensional stratification integrating age, antithrombotic status, angiographic classification, and hematoma characteristics—with each recommendation explicitly labeled by level of evidence (A/B/C)—represents a hypothesis-generating framework that warrants prospective validation before incorporation into clinical decision-making, rather than a guideline for current practice.

Fifth, the overall safety profile of MMAE is favorable, with severe complications in approximately 2–3% of procedures. The most clinically significant risks—procedure-related stroke, facial nerve injury, and rarely visual complications—should be systematically discussed with patients as part of shared decision-making.

The disease burden of cSDH is growing rapidly. MMAE, as one of the first minimally invasive treatments directly targeting the pathophysiological mechanisms of cSDH, represents an important therapeutic paradigm shift in this field. Omura and Ishiguro (47), in a systematic review of MMAE studies published before 2023 (2,783 cases), reported an MMAE-group recurrence rate of 6.3% (95% CI, 4.5–8.8%)—this figure should be understood as a summary description of prior clinical experience, constrained by the considerable heterogeneity in embolization techniques, patient selection, and recurrence definitions across the included studies, rather than as a basis for direct comparison with RCT data or with historical surgical data. Future research should pivot from the general question of “whether MMAE is effective” to the more precise question of “in which patients, using which strategy, at which time point, and in combination with which other treatments, MMAE can deliver optimal clinical value.” Answering this question requires not more traditional aggregate-data meta-analyses, but carefully designed, prospective, biomarker- and imaging-stratified, randomized trials—ideally underpinned by a standardized core outcome set and a collaborative individual patient data meta-analysis of the four completed landmark trials (61).
